# The role of personal risk factors in the occurrence of the hand-arm vibration syndrome: a pooled analysis of individual data from Italian cross-sectional and cohort studies

**DOI:** 10.1007/s00420-025-02169-0

**Published:** 2025-09-09

**Authors:** Massimo Bovenzi, Federico Ronchese, Francesca Larese Filon, Enrico Marchetti, Angelo Tirabasso, Carlotta Massotti, Marco Tarabini

**Affiliations:** 1 Clinical Unit of Occupational Medicine, Department of Medical Sciences, University of Trieste, U.C.O. di Medicina del Lavoro, Via Della Pietà 2/2, 34129 Trieste, Italy; 2 Department of Occupational and Environmental Medicine, Epidemiology and Hygiene, National Institute for Insurance Against Accidents at Work (INAIL), Monte Porzio Catone, Rome, Italy; 3 Department of Mechanical Engineering, Politecnico di Milano, Milan, Italy

**Keywords:** Exposure–response relationship, Hand-arm vibration syndrome, Hand-transmitted vibration, Personal risk factors, Pooled epidemiological studies, Upper limb disorders

## Abstract

**Purpose:**

To investigate the role of personal risk factors in the occurrence of the vascular, neurological and fibroproliferative disorders of the hand-arm vibration syndrome (HAVS) in workers groups exposed to hand-transmitted vibration (HTV).

**Methods:**

HAVS prevalence and incidence data were pooled across a series of cross-sectional studies (total sample: 1272 HTV workers, 579 controls) and prospective cohort studies (total sample: 377 HTV workers, 138 controls) conducted in Central and North-Eastern Italy. The pooled studies included detailed individual-level information about HTV exposure, personal risk factors, medical comorbidities and HAVS disorders. Vibration exposures were evaluated according to the recommendations of ISO standards.

**Results:**

The pooled studies revealed dose–response relationships between HTV exposure and the vascular and neurological components of HAVS. Older age, excessive alcohol intake, and comorbid conditions such as metabolic disorders, traumas/surgery to the neck and upper limbs, and disorders of the cervical spine were differentially associated with HAVS outcomes. Higher BMI had a protective effect on vascular disorders. Data modelling showed no significant interactions between HTV exposure and personal risk factors in the occurrence of upper limb disorders.

**Conclusions:**

The pooled analysis of epidemiological studies with individualised work, personal, and medical data confirmed that HTV exposure is a primary occupational risk factor for disorders in the fingers and hands of users of vibratory tools. Ageing and some personal factors connected to lifestyles and comorbidities were associated with an increased risk for upper limb disorders in HTV workers. Occupational and personal risk factors tended to contribute independently of each other to adverse outcomes in operators of hand-held vibrating machinery.

## Introduction

The hand-arm vibration syndrome (HAVS) is a collection of symptoms and signs of vascular, neurological, and musculoskeletal disorders occurring in the upper limbs of users of vibratory tools (CEN 1996). The vascular component of the HAVS is a secondary form of Raynaud’s phenomenon (RF) also known as vibration-induced white finger (VWF). The neurological component is characterised by a sensory neuropathy involving the territories innervated by the median nerve and, less frequently, the ulnar nerve. The musculoskeletal component includes degenerative and/or inflammatory changes in the hard and soft tissues of the upper extremities. The associations between these disorders and exposures to hand-transmitted vibration (HTV) are well established and some forms of exposure–response relationship have been reported, mainly for VWF (Griffin et al. 2003; Nilsson et al. 2017). However, upper limb disorders are also experienced by many people of the general population and are attributed, in addition to work activities, to a variety of personal risk factors or comorbidities which could act as confounders of the association between occupational exposures and health outcomes. In epidemiological studies, some methods are used to prevent bias of confounding, among which (i) exclusion from data analysis of individuals affected with comorbidities possibly associated with the outcomes studied, (ii) stratification by confounding factors, (iii) multivariable modelling with inclusion of confounders in the list of covariates so as to adjust the risk estimates for occupational exposures. In epidemiological studies of HTV related disorders, the focus is the relationship between the components of the HAVS and measures of daily or cumulative vibration exposures while the risk estimates for individual factors are rarely reported. In this study the individual, medical and occupational data of HTV workers investigated in a series of cross-sectional and cohort studies conducted in Italy were pooled and processed to estimate the possible contribution of personal risk factors to the occurrence of disorders associated with HTV exposure. Data pooling was considered feasible since the same surveying tools were used to investigate personal characteristics and health outcomes, uniform criteria were adopted for the definition of case affected with vascular and/or neurological disorders of the upper limbs, and the methods and procedures recommended by ISO standards were applied for the measurement of tool vibration and the evaluation of vibration exposures.

## Subjects and methods

### Pooled cross-sectional study

The study populations included 1272 HTV exposed male workers and 579 unexposed control men who were investigated in a series of cross-sectional studies conducted in geographic areas of the Central and North-Eastern Italy during the autumn/winter seasons in the calendar period 1990–2010 (Bovenzi 1998; Bovenzi et al. 1995, 2004, 2011b). Data from two additional epidemiological surveys of forestry workers undergoing compulsory health surveillance (unpublished) were added to the pooled cross-sectional database. The HTV exposed workers were employed in various industrial sectors: forestry (n = 524), construction (n = 221), shipbuiding (n = 192), engineering (n = 206), iron and steel (n = 129). The control men were manual workers (n = 429) or inspectors/supervisors (n = 150) not exposed to HTV and recruited in the same enterprises or companies of the HTV exposed workers.

### Pooled cohort study

The findings of two prospective cohort studies of HTV exposed male workers and unexposed control men were pooled in a longitudinal database (Bovenzi 2008, 2010; Bovenzi et al. 2011a, 2015). The cohort studies were conducted in Tuscany in the context ot the EU research projects VINET (2001) and VIBRISKS (2007). The cohorts included 377 HTV exposed workers (343 forestry operators and 34 stone workers) and 138 control men employed at the same companies (129 maintenance operators, 5 inspectors, 4 supervisors). They were investigated at the cross-sectional survey and over either 1 or 5 yr-interval follow-up investigations carried out in the autumn–winter seasons of the calendar period 1990–2007. Of the HTV exposed workers, 177 participated in three follow-ups (46.9%), 64 in two follow-ups (17%), and 136 in one follow-up survey (36.1%). Of the controls, 99 participated in three follow-ups (71.7%), 19 in two follow-ups (13.8%), and 20 in one follow-up survey (14.5%).

### Questionnaire and medical investigations

The HTV workers and the control men were interviewed by trained personnel on their personal, work, and health histories using a structured questionnaire initially developed by a team of occupational health professionals (Bovenzi et al. 1988) and then validated within the EU research projects VINET (2001) and VIBRISKS (2007). Following the medical interview, a physical examination focused on the vascular, neurological, and musculoskeletal systems of the neck and upper limbs was carried out by certified occupational physicians.

All subjects gave signed informed consent to participate in the original epidemiological studies, which were approved by the local health authorities of the NHS.

#### Personal factors

All studies collected information on age, height, weight, body mass index (BMI), ethnicity, education, marital status, smoking and drinking habits, and comorbid medical conditions. Cigarette smoking and alcohol consumption were expressed in terms of pack-years [(cigarettes/day/20) × years smoked] and units of alcohol per day (1 unit = 12 g of ethanol), respectively. The medical interview investigated previous and current personal health conditions by means of a series of questions concerning: (i) daily intake of medicines (e.g. vasodilators, β-blockers, analgesics, steroids, NSAIDs); (ii) previous traumas to the neck and/or upper limbs (home accidents, sport or occupational injuries); (iii) surgery to the neck and/or upper limbs; (iv) disorders of the cervical spine (positive clinical history substantiated by diagnostic imaging findings when available); (v) metabolic disorders (diabetes, arterial hypertension, overweight/obesity, metabolic syndrome, lipid or purine metabolism disorders); (vi) cardiovascular disorders (valvular or coronary heart diseases, arrhythmias, vascular diseases of the upper or lower limbs); (vii) other morbidities (e.g. migraine, previous Guillain-Barrè syndrome, thyroid diseases, connective tissue diseases).

#### HAVS outcomes

The anamnestic diagnosis of VWF at the medical interview was made according to the following criteria which were then established at the Stockholm Workshop’94 (Olsen et al. 1995): (i) positive history of cold provoked episodes of well-demarcated blanching in one or more fingers, (ii) first appearance of finger blanching after the start of occupational exposure to HTV, and (iii) experience of finger blanching attacks during the last 2 years. In the control men the anamnestic diagnosis of primary RF was made according to the classic criteria established by Allen and Brown (1932).

Sensorineural (SN) disorders were defined as *persistent* numbness (deteriorated cutaneous perception and/or loss of sensation) in the fingers and hands associated with impaired touch, temperature and vibrotactile perception, as well as difficulty in manipulating small objects, as observed at the traditional neurological examination. Case definition did not include subjects who reported temporary presence of tingling or numbness occurring while or after working with vibratory tools, during or following episodes of VWF or exposures to cold environment.

In the VIBRISKS cohort study (Bovenzi et al. 2015), the diagnosis of suspected carpal tunnel syndrome (CTS) was made according to the consensus criteria for the classification of CTS symptoms/signs in epidemiologic studies (Rempel et al. 1998). All the following criteria were required for the "clinical suspicion" of CTS: (i) classic/probable symptoms (numbness, tingling, burning or pain in at least two of digits 1, 2 or 3); (ii) nocturnal symptoms; and (iii) positive physical examination (Tinel’s test or Phalen’s test). In a limited number of cases, the diagnosis of CTS was established on the basis of either previous surgery for CTS or reports of abnormal electroneurographic findings suggestive for CTS.

In the pooled cross-sectional study, Dupuytren’s contracture (DC), a fibroproliferative connective tissue disorder of the palmar surface of the hands, could be investigated in the forestry worker group (n = 515, 98.3%) and about half of the controls (n = 288, 49.7%). In the pooled cohort study, all HTV workers and controls were examined for DC. The diagnosis of DC was based on the presence of nodules and cords in the palmar aponeurosis eventually resulting in irreversible flexion contracture of one or more fingers of the hand.

#### Cold provocation test

The cold test was performed with the subject in a supine position after a rest period of 20–30 min in a laboratory room with an air temperature of 20–22 °C. The measurement of finger circulation and the testing procedures were according to the finger cooling method suggested by Nielsen and Lassen (1977) and then recommended by the international standard ISO 14835–2 (2005). Briefly, the percentage change in finger systolic blood pressure (FSBP) from 30 to 10 °C (%FSBP_10°_) in a test finger (FSBP_t_), corrected for the change in systolic pressure in a reference finger (FSBP_ref_) of the same hand, was calculated as:

$$ {\text{\% FSBP}}_{{{10}^{ \circ } }} { = }\frac{{{\text{(FSBP}}_{{{\text{t,10}}^{ \circ } }} \times 100)}}{{\left[ {{\text{FSBP}}_{{{\text{t,30}}^{ \circ } }} - ({\text{FSBP}}_{{{\text{ref,30}}^{ \circ } }} - {\text{FSBP}}_{{{\text{ref,10}}^{ \circ } }} )} \right]}}(\% ) $$

Owing to organisational difficulties due to the availability of the measuring instrumentation in the field, the cold test could be carried out in 434 controls and 1070 HTV workers of the pooled cross-sectional study, corresponding to 75% and 84.1% of the surveyed populations. However, all HTV workers affected with VWF underwent the cold test. In the cohort studies, all subjects underwent the cold test approximately in the same calendar period (± 2 weeks) and FSBPs were measured in the same test and reference fingers at the various investigations.

## Measurement and assessment of vibration exposure

Vibration measurements were performed on the most representative hand-held tools used by the workers at the workplace. Vibration was measured on rotary, percussive, drilling or wrenching power tools used in metal-working, demolition, road construction and stone working, as well as on chain saws and brush saws used in forest work. Vibration measurements were made in the field during real operating conditions performed by skilled workers. The root-sum-of-squares (also called “vibration total value”, *a*_hv_) of the frequency weighted r.m.s. accelerations of tool *i* in three orthogonal directions (*x*, *y*, *z*) was calculated according to the international standard ISO 5349 (1986) afterwards replaced by ISO 5349–1 (2001):

$${a}_{\text{hvi}}= \sqrt{{a}_{\text{hwxi}}^{2}+{a}_{\text{hwyi}}^{2}+{a}_{\text{hwzi}}^{2}} ({\text{ms}}^{-2} \text{r}.\text{m}.\text{s}.)$$

In the pooled studies, the values of *a*_hv_ ranged from 2.7 to 18.3 ms^−2^ r.m.s. in the manufacturing industry (foundry work, shipbuilding and repairing), from 1.2 to 10.3 ms^−2^ r.m.s. in mechanical engineering, from 4 to 19.4 ms^−2^ r.m.s. in construction industry, from 9 to 23.1 ms^−2^ r.m.s. in stone working, and from 2.9 to 9.8 ms^−2^ r.m.s. in forest work (Bovenzi 1998; Bovenzi et al. 1995, 2004, 2011a).

Several information sources were used to estimate the duration of daily vibration exposure: workplace assessment questionnaires validated by interviews with employers and employees, examination of employment records, and direct observation of the work operations using a stopwatch method.

Daily vibration exposure was expressed in terms of frequency weighted r.m.s. acceleration magnitude normalised to an 8-h day (*A*(8)), according to the British Standard BS 6842 (1987) and the international standard ISO 5349–1 (2001):

$$A(8)=\sqrt{\sum_{i=1}^{n}{a}_{\text{hvi}}^{2}\frac{{T}_{i}}{{T}_{0}}} \left({\text{ms}}^{-2} \text{r}.\text{m}.\text{s}.\right)$$

where *a*_hv_ is the vibration total value of the r.m.s. acceleration of tool *i*, *T*_i_ is the duration of the *i*^th^ operation with tool *i* in hours, and *T*_0_ is the reference period of 8 h.

The use of vibratory tools during leisure time was reported by a limited number of forestry workers who operated occasionally chain or brush saws. However, the contribution of leisure activities to the individual work-related *A*(8) values was negligible.

## Ergonomic risk factors

To assess the possible association between suspected CTS and adverse ergonomic factors, in the VIBRISKS cohort study physical workload on the hands and forearms was investigated by means of five questions concerning twisting, forceful or repetitive movements, uncomfortable hand positions/grips, and heavy demands on precision scored on a 4-point response scale (VIBRISKS 2007; Bovenzi et al. 2015). The score of hand/forearm physical load was categorised into quartiles, which were assumed to correspond to four grades of increasing physical load: score 0–3 = no or mild load grade, score 4–6 = moderate load grade, score 7–9 = medium load grade, score 10–15 = hard load grade.

## Data analysis

The statistical analysis of data was performed with the Stata software, v. 18.0 (Stata Corporation, 2023). Continuous data were summarised with the median as a measure of central tendency and quartiles as measures of dispersion. Comparisons between independent groups were made with non-parametric statistics. The difference between categorical data tabulated in 2 × *k* contingency tables was tested by the *χ*^2^ test or the Fisher’ exact test. Point prevalence and cumulative incidence were calculated by conventional epidemiological methods.

In the pooled cross-sectional study, unconditional logistic regression analysis was used to assess the association between binary outcomes (VWF, SN, and DC disorders) and personal and occupational risk factors which entered the logistic models as continuous, dichotomous or tertile/quartile based design variables. Odds ratios (OR) and 95% confidence intervals (95% CI) were obtained from the estimated logistic regression coefficients and their robust standard errors. The significance of additional variables in the logistic models was assessed by the likelihood ratio *χ*^2^ test. The goodness-of-fit of the logistic models was assessed by the Hosmer–Lemeshow *χ*^2^ statistic.

In the pooled cohort study, the relations of outcomes to personal and occupational risk factors were assessed by the generalised estimating equations (GEE) method specifying an autoregressive correlation structure of the repeated measures of the individual’s response variables (Cui and Qian 2007). Continuous or binary response variables were modelled using identity or logit link functions, respectively. Both exposure variables and personal factors entered the linear or logistic models as time-dependent covariates, except for age at entry which was included as a time-independent variable.

In the pooled studies, interaction between explanatory variables was assessed by the inclusion of appropriate product terms.

Data analysis was performed on either the entire study populations or the HTV worker groups after exclusion of the control men.

A *P*-value of 0.05 was chosen as the limit of statistical significance.

## Results

### Characteristics of the study populations

At baseline the HTV workers differed significantly from the controls with respect to age, BMI and smoking and drinking habits in the pooled cross-sectional and cohort studies (Table 1). There were no significant differences in ethnicity (Caucasians *vs* non-Caucasians), marital status (married *vs* non-married), and education level (secondary school, less *vs* above) between HTV workers and controls (*P* = 0.20–0.50). Compared to the controls, metabolic disorders were reported more frequently by the HTV workers in both pooled studies, while disorders of the cervical spine, traumas or surgery to the neck and/or upper limbs, or cardiovascular disorders were more prevalent among the HTV workers in either of the pooled studies. The median (quartiles) values of daily vibration exposure expressed in terms of *A*(8) were 2.98 (1.90–4.41) ms^−2^ r.m.s. in the HTV workers of the pooled cross-sectional study and 3.72 (2.80–4.98) ms^−2^ r.m.s. in those of the pooled cohort study.

**Table 1:** Baseline characteristics of the study populations of the pooled cross-sectional and cohort studies

Pooled cross-sectional study				Pooled cohort study			
Factors	Controls (n = 579)	HTV workers (n = 1272)	Total sample (n = 1851)	Factors	Controls (n = 138)	HTV workers (n = 377)	Total sample (n = 515)
*Age (yr)*				*Age (yr)*			
20–37 (q1)	211 (36.4)	258 (20.3)	469 (25.3)	22–36 (q1)	49 (35.5)	100 (26.5)	149 (28.9)
37.1–45 (q2)	131 (22.6)	326 (25.6)	457 (24.7)	36.1–43 (q2)	45 (32.6)	90 (23.9)	135 (26.2)
45.1–52 (q3)	127 (21.9)	366 (28.8)	493 (26.6)	43.1–50 (q3)	22 (15.9)	100 (26.5)	122 (23.7)
52.1–73 (q4)	110 (19.0)	322 (25.3)^c^	432 (23.3)	50.1–69 (q4)	22 (15.9)	87 (23.1)^b^	109 (21.2)
*BMI (kg/m*^*2*^*)*				*BMI (kg/m*^*2*^*)*			
16.2–23.5 (q1)	177 (30.6)	288 (22.6)	465 (25.1)	18.4–23.5 (q1)	48 (34.8)	91 (24.1)	139 (27.0)
23.6–25.8 (q2)	153 (26.4)	308 (24.2)	461 (24.9)	23.6–25.6 (q2)	40 (29.0)	76 (20.2)	116 (22.5)
25.9–28.0 (q3)	133 (23.0)	332 (26.1)	465 (25.1)	25.7–27.7 (q3)	22 (15.9)	116 (30.8)	138 (26.8)
28.1–42.1 (q4)	116 (20.0)	344 (27.0)^c^	460 (24.9)	27.8–42.4 (q4)	28 (20.3)	94 (24.9)^c^	122 (23.7)
*Smoking (pack-yrs)*				*Smoking (pack-yrs)*			
Nonsmoker	313 (54.1)	562 (44.2)	875 (47.3)	Nonsmoker	89 (64.5)	121 (32.1)	210 (40.8)
0.1–11 (q1)	97(16.7)	231 (18.2)	328 (17.7)	0.2–10 (q1)	23 (16.7)	77 (20.4)	100 (19.4)
12–23 (q2)	83 (14.3)	247 (19.4)	330 (17.8)	11–22 (q2)	16 (11.6)	85 (22.6)	101 (19.6)
24–120 (q3)	86 (14.9)	232 (18.2)^c^	318 (17.2)	23–90 (q3)	10 (7.3)	94 (24.9)^c^	104 (20.2)
*Alcohol consumption (units/day)*				*Alcohol consumption (units/day)*			
Nondrinker	135 (23.3)	330 (25.9)	465 (25.1)	Nondrinker	34 (24.6)	95 (25.2)	129 (25.1)
1	122 (21.1)	195 (15.3)	317 (17.1)	1	63 (45.7)	85 (22.6)	148 (28.7)
2	138 (23.8)	290 (22.8)	428 (23.1)	2	29 (21.0)	80 (21.2)	109 (21.2)
≥ 3	184 (31.8)	457 (34.9)^a^	641 (34.6)	≥ 3	12 (8.7)	117 (31.0)^c^	129 (25.0)
Daily intake of medicines	85 (14.6)	228 (17.9)	313 (16.9)	Daily intake of medicines	24 (17.4)	50 (13.3)	74 (14.4)
Trauma/surgery to neck-upper limbs	239 (41.3)	631 (49.6)^c^	870 (47.0)	Trauma/surgery to neck-upper limbs	58 (42.0)	157 (41.6)	215 (41.8)
Disorders of the cervical spine	28 (4.9)	83 (6.5)	111 (6.0)	Disorders of the cervical spine	6 (4.4)	38 (10.1)^a^	44 (8.5)
Metabolic disorders	12 (2.1)	55 (4.3)^a^	67 (3.6)	Metabolic disorders	4 (2.9)	36 (9.6)^a^	40 (7.8)
Cardiovascular disorders	64 (11.1)	197 (15.5)^a^	261 (14.1)	Cardiovascular disorders	14 (10.1)	55 (14.6)	69 (13.4)
Duration of HTV exposure (yr)	–	18 (10–26)	–	Duration of HTV exposure (yr)	–	13 (7–20)	–
Daily exposure to HTV (hr)	–	2 (1–3.6)	–	Daily exposure to HTV (hr)	–	2.4 (1.4–3.3)	–
*A(8) (ms*^*−2*^* r.m.s.)*				*A(8) (ms*^*−2*^* r.m.s.)*			
0.30–1.90 (q1)	–	318 (25.0)	–	0.65–2.75 (q1)	–	88 (23.4)	–
1.91–2.98 (q2)	–	323 (25.4)	–	2.76–3.63 (q2)	–	86 (22.8)	–
2.99–4.41 (q3)	–	313 (24.6)	–	3.63–5.04 (q3)	–	113 (29.9)	–
4.42–19.3 (q4)	–	318 (25.0)	–	5.05–19.6 (q4)	–	90 (23.9)	–

Occupational and personal factors are expressed as continuous, binary, or tertile/quartile-based design variables (q). Data are given as medians (quartiles) or numbers (%)

*HTV* Hand-transmitted vibration

See text for the definition of *A*(8)

χ^2^ test (HTV workers *vs* controls): ^a^*P* < 0.05; ^b^*P* < 0.01; ^c^*P* < 0.001

### Occurrence of the disorders of HAVS

**Fig. 1:**
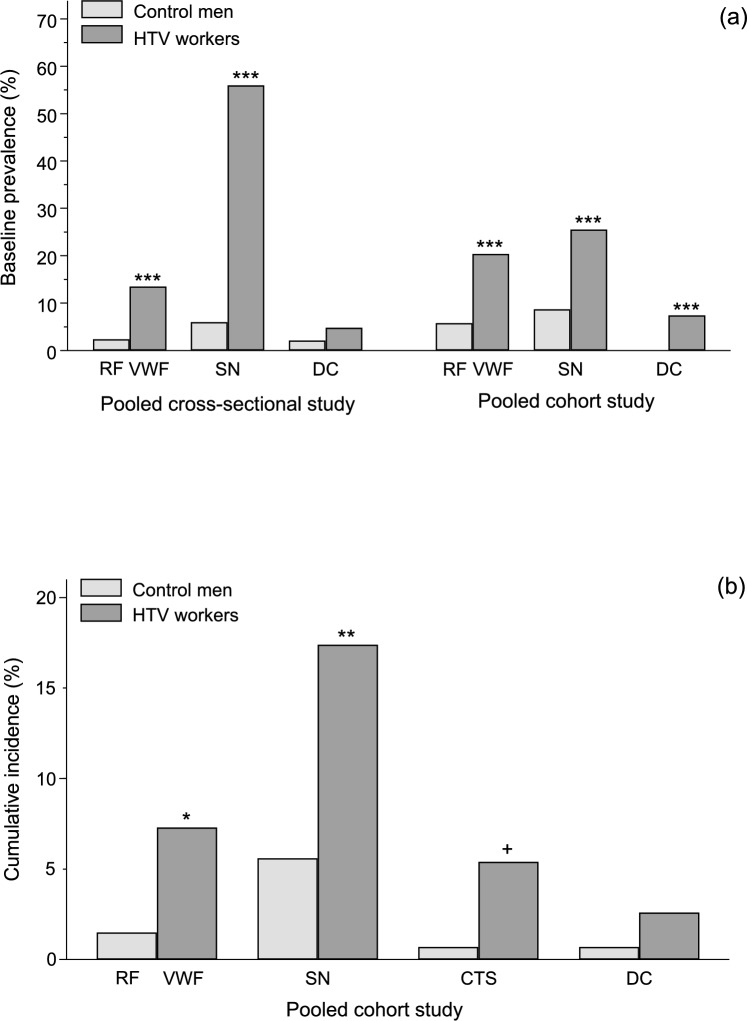
**a** Baseline prevalence of vascular disorders (RF^a^, VWF^b^), sensorineural (SN) disorders, and Dupuytren’s contracture (DC) in the control men and the workers exposed to hand-transmitted vibration (HTV) of the pooled cross-sectional and cohort studies. **b** Cumulative incidence of vascular disorders (RF^a^, VWF^b^), sensorineural (SN) disorders, carpal tunnel syndrome (CTS), and Dupuytren’s contracture (DC) in the control men and the workers exposed to hand-transmitted vibration (HTV) of the pooled cohort study. ^a^Raynaud’s phenomenon (RF) in the control men ^b^Vibration induced white finger (VWF) in the HTV workers χ^2^ test or Fisher’s exact test: ^+^*P* = 0.036; *P = 0.019; **P = 0.002; ***P < 0.001

In the two pooled studies, the baseline crude prevalences of VWF (RF in the controls), SN disorders, and DC were greater in the HTV workers than in the controls (Fig. 1a). In the pooled cohort study, the cumulative incidences for the HAVS disorders were greater in the HTV workers than in the controls, although not significantly for DC (Fig. 1b).

### Relations of the disorders of HAVS to occupational and personal risk factors

In the two pooled studies, age and occupational exposure to HTV were significant predictors of the probability of occurrence of the several components of the HAVS (Tables 2, 4, 5 and 6). After exclusion of the controls from data analysis, trends of increasing occurrence of VWF (Table 2), SN disorders (Table 4), and CTS (Table 5) with the increase in *A*(8) were observed in the HTV workers, suggesting quantitative dose–response relationships for HTV-related disorders of the hand-arm system. Hand/forearm physical load showed some elements of positive relation to CTS, but the association was not significant (*P* = 0.053–0.269). In both pooled studies DC was associated with HTV exposure but among the HTV workers there was no evidence for a trend of increasing DC risk with the increase in *A*(8) (Table 6).

**Table 2:** Relations of vibration induced white finger (VWF*) to vibration exposure and personal risk factors, expressed as continuous, binary, or tertile/quartile-based variables (q), in the entire study populations and the workers exposed to hand-transmitted vibration (HTV)

Factors	Pooled cross-sectional study		Pooled cohort study	
	Total sample (n = 1851)	HTV workers (n = 1272)	Total sample (n = 515, obs = 1665)	HTV workers (n = 377, obs = 1172)
	aOR (95% CI)	aOR (95% CI)	aOR (95% CI)	aOR (95% CI)
*Age (yr)*				
q1	1.0	1.0	1.0	1.0
q2	1.24 (0.58–2.64)	1.35 (0.75–2.43)	1.18 (0.74–1.87)	1.24 (0.79–1.94)
q3	**2.25 (1.01–5.02)**^**a**^	**3.26 (1.85–5.75)**^**c**^	1.64 (0.97–2.74)	**1.69 (1.02–2.81)**^**a**^
q4	**7.48 (3.27–17.1)**^**c**^	**3.50 (1.97–6.25)**^**c**^	**2.91 (1.68–5.03)**^**c**^	**2.62 (1.53–4.50)**^**c**^
Continuous (× 10 yr)	**1.72 (1.27–2.30)**^**b**^	**2.20 (1.56–3.10)**^**c**^	**1.83 (1.45–2.31)**^**c**^	**2.16 (1.68–2.78)**^**c**^
*BMI (kg/m*^*2*^*)*				
q1	1.0	1.0	1.0	1.0
q2	**0.40 (0.20–0.79)**^**b**^	0.75 (0.49–1.18)	0.90 (0.63–1.28)	0.92 (0.66–1.29)
q3	**0.24 (0.11–0.49)**^**c**^	**0.51 (0.32–0.82)**^**b**^	0.68 (0.45–1.02)	0.76 (0.52–1.11)
q4	**0.32 (0.15–0.68)**^**b**^	**0.29 (0.18–0.49)**^**c**^	0.65 (0.41–1.02)	0.75 (0.48–1.16)
Continuous	**0.89 (0.83–0.97)**^**b**^	**0.88 (0.84–0.93)**^**c**^	0.95 (0.89–1.00)	0.97 (0.90–1.03)
*Smoking (pack-yrs)*				
Nonsmoker	1.0	1.0	1.0	1.0
q1	1.61 (0.81–3.23)	1.13 (0.69–1.85)	1.41 (0.80–2.50)	1.38 (0.76–2.50)
q2	1.24 (0.59–2.60)	1.28 (0.82–1.99)	1.00 (0.57–1.75)	0.93 (0.52–1.65)
q3	1.27 (0.63–2.57)	1.16 (0.73–1.84)	1.15 (0.66–2.00)	1.11 (0.62–1.96)
*Alcohol consumption (units/day)*				
Nondrinker	1.0	1.0	1.0	1.0
1	1.73 (0.82–3.66)	1.04 (0.60–1.82)	1.14 (0.74–1.75)	1.00 (0.66–1.50)
2	1.22 (0.54–2.76)	0.83 (0.50–1.37)	1.39 (0.92–2.11)	1.32 (0.90–1.94)
≥ 3	1.65 (0.76–3.57)	1.26 (0.83–1.92)	1.41 (0.90–2.19)	1.25 (0.83–1.88)
*A(8) (ms*^*−2*^* r.m.s.)*				
Controls	1.0	–	1.0	–
q1	1.59 (0.41–6.18)	1.0	**2.75 (1.25–6.05)**^**b**^	1.0
q2	1.39 (0.48–4.03)	1.40 (0.80–2.44)	**3.98 (1.87–8.49)**^**c**^	1.35 (0.91–2.00)
q3	**2.96 (1.42–6.17)**^**b**^	**2.66 (1.59–4.45)**^**c**^	**4.04 (1.89–8.61)**^**c**^	1.30 (0.84–2.02)
q4	**4.54 (2.22–9.32)**^**c**^	**3.76 (2.26–6.27) c**	**5.27 (2.39–11.6)**^**c**^	**1.70 (1.00–2.91)**^**a**^
Continuous	–	**1.17 (1.09–1.26)**^**c**^	–	**1.20 (1.12–1.29)**^**c**^
Exposure to HTV (yes *vs* no)	**3.02 (1.59–5.73)**^**c**^	–	**3.91 (1.86–8.23)**^**c**^	–
Daily intake of medicines	0.67 (0.28–1.60)	0.51 (0.17–1.52)	1.13 (0.77–1.65)	1.24 (0.88–1.75)
Trauma/surgery to neck-upper limbs	0.54 (0.28–1.03)	0.51 (0.28–1.03)	0.93 (0.63–1.36)	0.88 (0.60–1.29)
Disorders of the cervical spine	1.10 (0.45–2.72)	0.85 (0.32–2.29)	1.13 (0.60–2.15)	1.14 (0.58–2.23)
Metabolic disorders	**3.09 (1.11–8.58)**^**a**^	**3.43 (1.07–10.9)**^**a**^	**2.27 (1.19–4.35)**^**a**^	**2.43 (1.23–4.82)**^**b**^
Cardiovascular disorders	1.98 (0.87–4.49)	1.51 (0.55–4.11)	1.20 (0.73–1.98)	1.00 (0.61–1.63)

Adjusted odds ratios (aOR) and robust 95% confidence intervals (95% CI) are estimated by means of multivariable logistic regression in the pooled cross-sectional study and the generalised estimating equations method with a logit link in the pooled cohort study. Significant associations in bold

^*^Raynaud’s phenomenon in the controls

See text for the definition of *A*(8)

See Table 1 for the definition of quantile (q) cut-points

^a^*P* < 0.05; ^b^*P* < 0.01; ^c^*P* < 0.001

On investigating the influence of personal risk factors on the occurrence of HAVS disorders, vascular symptoms were significantly associated with metabolic disorders in both pooled studies, while inverse relations were observed for BMI with a decreasing trend which was significant in the pooled cross-sectional study (Table 2). Logistic regression analysis revealed no significant first-order interactions between age, BMI, and metabolic disorders (*P* = 0.606–0.965).

In both pooled studies, cold-induced vasoconstriction (i.e. reduction of %FSBP_10°_) was associated with HTV exposure and vascular symptoms, while the digital vasoconstrictor response to cold was more attenuated with the increase in BMI (Table 3).

**Table 3:** Relations of the cold response of digital arteries (%FSBP_10°_) to vibration exposure and personal risk factors, expressed as binary or continuous variables, in the entire study population and the workers exposed to hand-transmitted vibration (HTV)

Factors	Pooled cross-sectional study		Pooled cohort study	
	Total sample (n = 1504)	HTV workers (n = 1070)	Total sample (n = 515, obs = 1665)	HTV workers (n = 377, obs = 1172)
	Coefficient (95% CI)	Coefficient (95% CI)	Coefficient (95% CI)	Coefficient (95% CI)
Age (× 10 yr)	0.05 ( − 1.97; 2.07)	− 0.53 (− 2.90; 1.83)	− 0.88 (− 2.14; 0.39)	**− 2.29 (− 3.86; − 0.72)**^**b**^
BMI (kg/m^2^)	**0.54 (0.005; 1.07)**^**a**^	0.47 (− 0.15; 1.09)	**0.67 (0.31; 1.03)**^**c**^	**0.74 (0.29; 1.18)**^**c**^
Smoking (yes *vs* no)	1.27 (− 2.75; 5.28)	1.86 (− 3.03; 6.76)	0.54 (− 1.70; 2.78)	1.08 (− 1.74; 3.91)
Drinking (yes *vs* no)	− 0.84 (-4.87; 3.18)	0.51 (− 4.52; 5.55)	− 1.42 (− 3.91; 1.07)	1.33 (− 1.83; 4.49)
Exposure to HTV (yes *vs* no)	**− 6.03 (− 9.62; − 2.44)**^**c**^	–	**− 6.30 (− 8.76; − 3.85)**^**c**^	–
*A*(8) (ms^−2^ r.m.s.)	–	**− 1.61 (− 2.54; − 0.68)**^**c**^	–	**− 2.14 (− 2.59; − 1.69)**^**c**^
VWF*	**− 18.1 (– 26.4; − 9.85)**^**c**^	**− 15.6 (– 24.5; − 6.75)**^**c**^	**− 19.3 (− 22.3; − 16.3)**^**c**^	**− 15.8 (− 19.3; − 12.2)**^**c**^
Daily intake of medicines	2.46 (− 4.07; 8.99)	4.11 (− 4.78; 13.0)	0.84 (− 2.21; 3.88)	3.29 (− 0.52; 7.11)
Trauma/surgery to neck-upper limbs	− 0.05 (− 3.76; 3.67)	0.97 (− 3.82; 5.76)	− 0.14 (− 2.27; 1.99)	0.25 (− 2.46; 2.95)
Disorders of the cervical spine	− 1.20 (− 9.76; 7.36)	2.44 (− 6.46; 11.3)	1.78 (− 2.41; 5.98)	4.32 (− 0.58; 9.23)
Metabolic disorders	− 7.43 (− 20.3; 5.46)	− 2.13 (− 17.0; 12.7)	− 0.13 (− 4.74; 4.47)	1.29 (− 4.00; 6.58)
Cardiovascular disorders	− 1.77 (− 9.16; 5.61)	− 0.54 (− 9.96; 8.88)	− 2.65 (− 6.34; 1.04)	− 3.41 (− 7.86; 1.05)
Intercept	79.2 (63.9; 94.6)	81.2 (61.4; 101)	81.0 (70.8; 91.1)	84.6 (71.4; 97.7)

Adjusted coefficients and robust 95% confidence intervals (95% CI) are estimated by means of multivariable linear regression in the pooled cross-sectional study and the generalised estimating equations method with an identity link in the pooled cohort study. Significant associations in bold

^*^VWF: vibration-induced white finger, Raynaud’s phenomenon in the controls

See text for the definition of *A*(8) and %FSBP_10°_

^a^*P* < 0.05; ^b^*P* < 0.01; ^*c*^*P* < 0.001

Linear and logistic regression analysis showed no significant associations between vascular disorders (symptoms and cold test results) and smoking or drinking habits in both the pooled cross-sectional and cohort studies.

In addition to age and HTV exposure, traumatic events and/or surgery to neck and upper limbs were significant determinants of the occurrence of SN disorders (Table 4) and CTS (Table 5) in both pooled studies. In the pooled cross-sectional study, disorders of the cervical spine were significantly associated with SN disorders. In the two pooled studies, alcohol consumption ≥ 3 units/day was predictive of the occurrence of CTS (Table 5) and DC (Table 6): in the pooled cohort study heavier drinkers exhibited about a threefold increased risk for CTS and DC compared to nondrinkers. No significant relations were found between the neurological components of the HAVS and smoking habit, metabolic and cardiovascular disorders in either pooled studies. In opposite, DC showed a strong association with metabolic disorders: in both pooled studies, the risk for DC was 4 to fivefold higher in the individuals with metabolic disorders compared to those not affected with metabolic dysfunctions.

**Table 4:** Relations of sensorineural (SN) disorders to vibration exposure and personal risk factors, expressed as binary, continuous, or tertile/quartile-based design variables (q), in the entire study populations and the workers exposed to hand-transmitted vibration (HTV)

Factors	Pooled cross-sectional study		Pooled cohort study	
	Total sample (n = 1851)	HTV workers (n = 1272)	Total sample (n = 515, obs = 1665)	HTV workers (n = 377, obs = 1172)
	aOR (95% CI)	aOR (95% CI)	aOR (95% CI)	aOR (95% CI)
*Age (yr)*				
q1	1.0	1.0	1.0	1.0
q2	1.28 (0.72–2.26)	1.25 (0.65–2.41)	1.21 (0.89–1.63)	1.09 (0.76–1.58)
q3	1.56 (0.84–2.88)	1.83 (0.92–3.66)	**1.79 (1.25–2.56)**^**c**^	**1.69 (1.11–2.58)**^**a**^
q4	**3.50 (1.78–6.87)**^**c**^	**3.71 (1.73–7.95)**^**c**^	**2.69 (1.81–4.02)**^**c**^	**2.65 (1.67–4.20)**^**c**^
Continuous (× 10 yr)	**1.53 (1.21–1.94)**^**c**^	**1.67 (1.28–2.18)**^**c**^	**1.92 (1.50–2.47)**^**c**^	**2.06 (1.58–2.69)**^**c**^
*BMI (kg/m*^*2*^*)*				
q1	1.0	1.0	1.0	1.0
q2	1.17 (0.66–2.10)	1.54 (0.79–3.00)	0.81 (0.63–1.05)	0.80 (0.59–1.08)
q3	1.59 (0.91–2.78)	**2.18 (1.15–4.17)**^**a**^	0.94 (0.71–1.27)	0.99 (0.71–1.39)
q4	1.20 (0.65–2.21)	1.25 (0.61–2.53)	1.12 (0.81–1.56)	1.14 (0.78–1.67)
Continuous	1.02 (0.97–1.07)	1.02 (0.93–1.06)	1.00 (0.95–1.05)	1.00 (0.94–1.06)
*Smoking (pack-yrs)*				
Nonsmoker	1.0	1.0	1.0	1.0
q1	1.38 (0.80–2.41)	1.13 (0.60–2.13)	1.24 (0.77–2.00)	1.11 (0.65–1.89)
q2	0.94 (0.53–1.66)	0.81 (0.43–1.53)	1.31 (0.84–2.05)	1.21 (0.74–1.99)
q3	1.50 (0.85–2.64)	1.51 (0.81–2.83)	1.35 (0.85–2.16)	1.29 (0.78–2.15)
*Alcohol consumption (units/day)*				
Nondrinker	1.0	1.0	1.0	1.0
1	1.60 (0.87–2.95)	1.69 (0.84–3.41)	1.15 (0.87–1.52)	1.25 (0.89–1.75)
2	1.71 (0.91–3.19)	1.92 (0.96–3.86)	1.16 (0.87–1.55)	1.19 (0.85–1.66)
≥ 3	1.84 (0.99–3.43)	1.87 (0.94–3.73)	0.72 (0.52–1.01)	0.82 (0.57–1.18)
*A(8) (ms*^*−2*^* r.m.s.)*				
Controls	1.0	–	1.0	–
q1	1.71 (0.58–5.07)	1.0	**2.14 (1.18–3.87)**^**b**^	1.0
q2	**2.57 (1.22–5.39)**^**a**^	2.27 (0.95–5.43)	**2.59 (1.45–4.61)**^**c**^	**1.22 (0.87–1.73)**
q3	**4.11 (2.30–7.35)**^**c**^	**2.44 (1.10–5.42)**^**a**^	**3.38 (1.91–6.00)**^**c**^	**1.65 (1.13–2.42)**^**b**^
q4	**3.98 (2.21–7.15)**^**c**^	**2.63 (1.18–5.86)**^**a**^	**3.26 (1.75–6.05)**^**c**^	**1.63 (1.01–2.65)**^**a**^
Continuous	–	**1.08 (1.01–1.16)**^**a**^	–	**1.11 (1.03–1.20)**^**b**^
Exposure to HTV (yes *vs* no)	**3.55 (2.12–5.95)**^**c**^	–	**2.87 (1.63–5.02)**^**c**^	–
Daily intake of medicines	1.13 (0.56–2.27)	1.25 (0.59–2.63)	0.91 (0.69–1.20)	0.88 (0.64–1.21)
Trauma/surgery to neck-upper limbs	**2.41 (1.60–3.61)**^**c**^	**1.99 (1.27–3.13)**^**b**^	**1.37 (1.00–1.87)**^**a**^	1.37 (0.97–1.93)
Disorders of the cervical spine	**3.45 (1.70–6.99)**^**c**^	**3.99 (1.75–9.11)**^**c**^	1.12 (0.65–1.92)	1.31 (0.72–2.38)
Metabolic disorders	1.63 (0.63–4.23)	2.03 (0.73–5.59)	1.23 (0.65–2.32)	1.37 (0.69–2.71)
Cardiovascular disorders	1.42 (0.71–2.86)	1.29 (0.61–2.74)	1.26 (0.85–1.86)	1.06 (0.68–1.64)

Adjusted odds ratios (aOR) and robust 95% confidence intervals (95% CI) are estimated by means of multivariable logistic regression in the pooled cross-sectional study and the generalised estimating equations method with a logit link in the pooled cohort study. Significant associations in bold

See text for the definition of *A*(8)

See Table 1 for the definition of quantile (q) cut-points

^*a*^*P* < 0.05; ^b^*P* < 0.01; ^c^*P* < 0.001

**Table 5:** Relations of suspected carpal tunnel sydrome to vibration exposure and personal risk factors, expressed as continuous, binary, or tertile/quartile-based design variables (q), in the entire study population and the workers exposed to hand-transmitted vibration (HTV) of the VIBRISKS cohort study

Factors	Pooled cohort study	
	Total sample (n = 387, obs = 1380)	HTV workers (n = 249, obs = 888)
	aOR (95% CI)	aOR (95% CI)
*Age (yr)*		
q1	1.0	1.0
q2	2.36 (0.92–6.06)	**2.77 (1.03–7.44)**^**a**^
q3	1.57 (0.59–6.21)	1.76 (0.63–4.92)
q4	**3.39 (1.26–9.14)**^**a**^	**3.52 (1.22–10.1)**^**a**^
Continuous (× 10 yr)	**1.63 (1.17–2.27)**^**b**^	**1.61 (1.15–2.27)**^**b**^
*BMI (kg/m*^*2*^*)*		
q1	1.0	1.0
q2	2.42 (0.88–6.68)	2.22 (0.79–6.26)
q3	2.63 (0.97–7.12)	2.43 (0.88–6.70)
q4	2.57 (0.93–7.08)	2.35 (0.84–6.59)
Continuous	1.06 (0.99–1.15)	1.08 (0.99–1.17)
*Smoking (pack-yrs)*		
Nonsmoker	1.0	1.0
q1	1.81 (0.85–3.89)	1.53 (0.69–3.42)
q2	0.93 (0.44–1.98)	0.89 (0.41–1.93)
q3	0.37 (0.11–1.19)	0.36 (0.10–1.24)
*Alcohol consumption (units/day)*		
Nondrinker	1.0	1.0
1	1.04 (0.44–2.49)	1.15 (0.46–2.88)
2	1.08 (0.46–2.53)	1.18 (0.48–2.92)
≥ 3	**2.97 (1.26–6.99)**^**a**^	**3.41 (1.37–8.44)**^**b**^
*A(8) (ms*^*−2*^* r.m.s.)*		
Controls	1.0	–
q1	**11.5 (2.85–46.6)**^**c**^	1.0
q2	**6.92 (1.57–30.6)**^**b**^	0.61 (0.21–1.75)
q3	**18.8 (4.86–73.0)**^**c**^	1.69 (0.73–3.89)
q4	**29.9 (7.80–115)**^**c**^	**2.66 (1.20–5.90)**^**a**^
Continuous	–	**1.12 (1.05–1.19)**^**b**^
Exposure to HTV (yes *vs* no)	**14.9 (4.12–54.3)**^**c**^	–
*Hand/forearm physical load*		
No/mild (score 0–3)	1.0	1.0
Moderate (score 4–6)	1.05 (0.41–2.65)	1.57 (0.51–4.85)
Medium (score 7–9)	0.51 (0.20–1.29)	0.70 (0.23–2.12)
Hard (score 10–15)	1.47 (0.61–3.57)	1.48 (0.50–4.36)
Continuous	1.09 (0.99–3.25)	1.05 (0.96–1.16)
Daily intake of medicines	1.74 (0.85–3.56)	1.93 (0.93–3.98)
Trauma/surgery to neck-upper limbs	**1.89 (1.05–3.42)**^**a**^	**1.87 (1.02–3.42)**^**a**^
Disorders of the cervical spine	1.70 (0.64–4.51)	2.05 (0.75–5.61)
Metabolic disorders	1.96 (0.47–8.27)	2.65 (0.58–12.1)
Cardiovascular disorders	1.09 (0.44–2.71)	0.83 (0.31–2.24)

Adjusted odds ratios (aOR) and robust 95% confidence intervals (95% CI) are estimated by means of the generalised estimating equations method with a logit link. Significant associations in bold

See text for the definition of *A*(8)

See Table 1 for the definition of quantile (q) cut-points

^a^*P* < 0.05; ^b^*P* < 0.01; ^c^*P* < 0.001

**Table 6:** Relations of Dupuytren’s contracture to vibration exposure and personal risk factors, expressed as continuous, binary, or tertile/quartile-based design variables (q), in the entire study populations and the workers exposed to hand-transmitted vibration (HTV)

Factors	Pooled cross-sectional study		Pooled cohort study	
	Total sample (n = 803)	HTV workers (n = 515)	Total sample (n = 515, obs = 1665)	HTV workers (n = 377, obs = 1172)
	aOR (95% CI)	aOR (95% CI)	aOR (95% CI)	aOR (95% CI)
*Age (yr)*				
q1	1.0	1.0	1.0	1.0
q2	0.78 (0.19–3.15)	0.86 (0.16–4.48)	0.80 (0.23–2.76)	0.80 (0.28–2.25)
q3	2.42 (0.66–8.88)	2.17 (0.35–13.3)	1.23 (0.39–3.86)	0.91 (0.31–2.66)
q4	3.53 (0.89–13.9)	3.29 (0.46–23.4)	**3.02 (1.02–8.91)**^**a**^	1.75 (0.61–5.03)
Continuous (× 10 yr)	**1.95 (1.17–3.25)**^**b**^	**1.84 (1.05–3.24)**^**a**^	**1.95 (1.28–2.99)**^**b**^	1.47 (0.97–2.22)
*BMI (kg/m*^*2*^*)*				
q1	1.0	1.0	1.0	1.0
q2	0.51 (0.15–1.68)	0.45 (0.11–1.77)	0.56 (0.22–1.42)	0.74 (0.31–1.74)
q3	0.89 (0.31–2.53)	0.62 (0.18–2.16)	1.20 (0.53–2.71)	1.42 (0.62–3.24)
q4	0.32 (0.08–1.27)	0.20 (0.04–1.11)	0.42 (0.16–1.12)	0.69 (0.26–1.80)
Continuous	0.99 (0.87–1.15)	0.93 (0.81–1.08)	0.97 (0.86–1.08)	1.00 (0.89–1.11)
*Smoking (pack-yrs)*				
Nonsmoker	1.0	1.0	1.0	1.0
q1	0.14 (0.02–1.09)	0.12 (0.01–1.09)	0.64 (0.24–1.69)	0.53 (0.18–1.57)
q2	0.92 (0.34–2.49)	0.54 (0.17–1.73)	0.63 (0.27–1.51)	0.58 (0.22–1.54)
q3	0.49 (0.16–1.50)	0.35 (0.09–1.37)	0.56 (0.23–1.33)	0.57 (0.21–1.54)
*Alcohol consumption (units/day)*				
Nondrinker	1.0	1.0	1.0	1.0
1	0.60 (0.13–2.82)	0.36 (0.06–2.10)	0.36 (0.08–1.61)	0.65 (0.19–2.25)
2	1.83 (0.50–6.75)	1.64 (0.44–6.14)	1.49 (0.59–3.72)	1.41 (0.56–3.53)
≥ 3	**3.49 (1.04–11.7)**^**a**^	2.24 (0.59–8.56)	**2.65 (1.02–6.04)**^**a**^	**2.72 (1.18–6.28)**^**a**^
*A(8) (ms*^*−2*^* r.m.s.)*				
Controls	1.0	–	1.0	–
q1	1.90 (0.33–10.9)	1.0	**33.1 (1.93–566)**^**a**^	1.0
q2	1.48 (0.33–6.58)	0.80 (0.11–5.60)	10.1 (0.56–183)	0.28 (0.11–1.05)
q3	1.92 (0.62–6.00)	1.04 (0.16–6.62)	**27.6 (1.60–477)**^**a**^	0.83 (0.39–1.79)
q4	2.18 (0.69–7.10)	1.25 (0.18–8.76)	**19.4 (1.05–359)**^**a**^	0.98 (0.38–2.54)
Continuous	–	0.97 (0.81–1.17)	–	0.96 (0.82–1.13)
Exposure to HTV (yes vs no)	2.00 (0.70–5.68)	–	**23.9 (3.05–188)**^**b**^	–
Daily intake of medicines	0.55 (0.12–2.43)	0.62 (0.16–2.36)	0.33 (0.11–1.02)	0.28 (0.08–1.10)
Trauma/surgery to neck-upper limbs	1.15 (0.51–2.60)	0.95 (0.35–2.54)	0.75 (0.38–1.46)	0.81 (0.39–1.68)
Disorders of the cervical spine	2.34 (0.72–7.57)	2.13 (0.55–8.30)	0.87 (0.32–2.38)	1.08 (0.36–3.24)
Metabolic disorders	4.12 (0.91–18.6)	**4.80 (1.11–20.8)**^**a**^	**4.76 (2.05–11.0)**^**c**^	**5.09 (1.98–13.0)**^**c**^
Cardiovascular disorders	1.60 (0.41–6.21)	2.08 (0.61–7.08)	1.70 (0.72–3.99)	1.72 (0.68–4.39)

Adjusted odds ratios (aOR) and robust 95% confidence intervals (95% CI) are estimated by means of multivariable logistic regression in the pooled cross-sectional study and the generalised estimating equations method with a logit link in pooled the cohort study. Significant associations in bold

See text for the definition of *A*(8)

See Table 1 for the definition of quantile (q) cut-points

^a^*P* < 0.05; ^*b*^*P* < 0.01; ^c^*P* < 0.001

Goodness-of-fit statistic revealed that logistic models of the pooled cross-sectional study fitted reasonably well the HAVS outcomes (*P* = 0.343–0.964).

Overall, there was no evidence of significant first-order interactions between daily exposure to HTV expressed as *A*(8) and individual characteristics or comorbidities in the study populations (*P* = 0.238–0.929 in the pooled cross-sectional study; *P* = 0.166–0.760 in the pooled cohort study). As an example, in the pooled cross-sectional study logistic regression analysis with VWF as the outcome (Table 2) showed that the *χ*^2^ statistic varied from 3.61 (*df* 7) for the interaction *A*(8) × age (*P* = 0.824) to 0.34 (*df* 2) for the interaction *A*(8) × metabolic disorders (*P* = 0.844). Similar non-significant interactions were observed in the pooled cohort study, although to a lesser extent: for the probability of VWF occurrence, the *χ*^2^ values were 10.6 (*df* 9) for the interaction *A*(8) × age (*P* = 0.300) and 5.08 (*df* 3) for the interaction *A*(8) × metabolic disorders (*P* = 0.167).

## Discussion

The present study aimed to understand the influence of individual risk factors on the occurrence of the HAVS disorders, as well as to investigate quantitatively the relative role and contribution of HTV exposure and personal risk factors to the development of upper limb disorders in professional users of hand-held power tools.

### Disorders of the upper limbs and HTV exposure

The pooled analysis of individual data of our epidemiological studies carried out over the last three decades confirms that occupational exposure to HTV treated as either a qualitative (binary) or a quantitative (*A*(8)) variable is a primary factor in the etiopathogenesis of the vascular and neurosensory disorders occurring in the upper limbs of workers operating vibratory tools or machines. These findings are consistent with those of a recent systematic review and meta-analysis of epidemiological studies with a low risk of bias which reported meta-OR of 6.85 (95% CI 4.17–11.25) for Raynaud’s phenomenon, 7.37 (95% CI 4.39–12.37) for neurosensory disorders, and 2.93 (95% CI 1.74–4.95) for CTS when workers exposed to HTV were compared with non-vibration exposed worker groups (Nilsson et al. 2017). Moreover, the findings of our pooled studies provided strong evidence for dose–response relationships between HTV exposure and VWF and SN disorders, confirming the results of previous cross-sectional and prospective cohort studies of HTV exposed workers employed in several industrial sectors (Griffin et al. 2003; Edlund et al. 2014). The findings of the two pooled studies are in accord with those of systematic reviews and meta-analyses of studies selected with high quality methodological criteria which have reported a more than twofold risk for CTS and DC associated with prolonged use of power machinery (Palmer et al. 2007; Mathieu et al. 2020; Nilsson et al. 2023).

### Vascular disorders of the HAVS and personal risk factors

In the two pooled studies, vascular symptoms of white finger were associated with metabolic disorders and showed an increasing trend of occurrence with the increase of age. It may be hypothesised that atherosclerotic changes in the digital vessels due to ageing and metabolic dysfunction, as well combined with the remodeling of the vessel wall caused by vibration induced arterial intimal and muscular layers thickening, can result in a reduction of the diameter of the arterial lumen which facilitates the closure phenomenon of the digital arteries triggered by cold exposure during a Raynaud’s attack (Takeuki et al. 1986; Reda et al. 2022).

In our study populations there was evidence for an inverse relationship between digital vasospastic symptoms and BMI. Consistently, the vasoconstrictive response of digital arteries to local cooling was lessened with the increase in BMI. Negative associations between the occurrence of Raynaud’s phenomenon and BMI have been observed in several population- or community-based epidemiological studies carried out in Europe and U.S. (Fraenkel 2002; Fraenkel et al. 1999; Palesch et al. 1999; Stjernbrandt et al. 2022). To our knowledge, our laboratory finding of an attenuated cold-induced digital arterial vasoconstriction in individuals with higher BMI has not been reported previously. The mechanisms underlying the protective effects of BMI on the risk of Raynaud’s phenomenon is unclear. It has been argued that either thinner persons may be more susceptible to cold exposure or overweighted or obese cases are protected by the insulating properties of subcutaneous fat (Fraenkel 2002; Stjernbrandt et al. 2022). Since the logistic models of our pooled studies showed no significant interactions between HTV exposure, age, BMI, and metabolic disorders, it may be argued that these explanatory variables are likely to act as independent determinants of the occurrence of vascular disorders in the study populations.

No significant associations were observed between vascular symptoms and alcohol or cigarette consumption in both pooled studies. Similarly, cold test results were not associated with smoking and drinking habits. Conflicting results are reported in epidemiological studies examining the association between smoking and primary Raynaud’s phenomenon or VWF. Positive associations have been interpreted on the basis of the vasoconstrictor effects of nicotine on the peripheral circulation (Fraenkel et al. 1999), but other investigators did not find an increased prevalence of RF or VWF in male smokers compared to non-smokers in epidemiological studies of either the general population or HTV exposed groups, respectively (Palesch et al. 1999; Bovenzi et al. 1994; Stjernbrandt et al. 2022). Alcohol consumption has been found to be associated with Raynaud’s phenomenon in women but not in men (Fraenkel et al. 1999). It has been suggested that hormonal factors could be the mediators of the excess risk in women since alcohol tend to increase the level of circulating estrogens (Fraenkel 2002). However, other studies failed to find any association between alcohol and Raynaud’s phenomenon in either sex (Palesch et al. 1999).

### Neurosensory disorders of the HAVS and personal risk factors

The occurrence of SN disorders were related to age and two comorbidities such as traumas or surgery to the neck or upper limbs and disorders of the cervical spine. These adverse health conditions may give rise to dysfunction and disruption of cutaneous mechanoreceptors and afferent sensory nerve fibres leading to impairment to tactile and temperature perception, numbness and loss of touch sensation. It has been reported that HTV exposure can provoke nerve injuries as revealed by histopathological studies of either experimental animals exposed to acute vibration or human biopsy specimens of nerves of the finger-hand-wrist segments. A variety of lesions have been observed in both myelinated and unmyelinated nerve fibres of HTV workers such as degeneration or loss of axons, demyelination and perineural fibrosis (Tacheuki et al. 1986; Lundborg et al. 1987; Strömberg et al. 1997). It is plausible that comorbidities in the neck and upper limbs can concur with occupational exposure to HTV to increase the occurrence of neurosensory disorders in professional users of vibrating tools.

Three non-occupational factors were significantly related to suspected CTS in the pooled cohort study: age, alcohol consumption ≥ 3 units/day, and traumatic events (accidental or surgical). There was no association with smoking habit and this is consistent with the results of a meta-analysis of cohort, case–control, and cross-sectional studies of high quality and adjusted for publication bias which did not support an association between CTS and current *vs* past/never smokers (Lampainen et al. 2022). Consistently with our findings, epidemiological studies of the general or working populations and meta-analyses have reported an increased risk for CTS with increasing age and alcohol consumption (Harris-Adamson et al. 2013). The role of overweight and obesity on the risk of developing CTS is controversial. Some studies have reported an increased risk for CTS with the increase in BMI, while other studies found no effect of BMI on CTS risk (Nordstrom et al. 1997; Shiri et al. 2015; Burger et al. 2016). In our pooled cohort study, BMI, treated as a continuous variable, showed a marginally not significant association with the occurrence of CTS; the risk of CTS increased by 6–8% for each one-unit of increase in BMI (kg/m^2^). These figures are remarkably similar to those found in a U.S. population based case–control study and a meta-analysis of 58 studies which reported that a one-unit increase in BMI was associated with 8% and 7.4% increased risk for CTS, respectively (Nordstrom et al. 1997; Shiri et al. 2015). At the light of the findings of the epidemiological literature, it is prevalent opinion among researchers that CTS is a disorder of multifactorial origin but the influence of the various individual factors on CTS risk is still debated.

### Dupuytren’s contracture and personal risk factors

In addition to HTV exposure, age, alcohol consumption ≥ 3 units/day, and metabolic disorders were significantly associated with the occurrence of DC in the pooled studies. Metabolic disorders was a strong predictor of DC risk, and among DC cases there were patients affected with dyslipidaemia, gout, type 2 diabetes mellitus treated with oral hypoglycaemic drugs, arterial hypertension, and obesity either isolated or combined in a diagnosis of metabolic syndrome. There were inverse, not significant, associations between DC and BMI in both pooled studies. A possible protective effect of BMI on the probability of DC development has been reported in some investigations, but other studies reported either an increased risk of DC with increasing BMI or a null effect (Geoghegan et al. 2004; Hacquebord et al. 2017). Overall, our findings are consistent with those of several epidemiological studies, narrative/systematic reviews and meta-analyses of the relations of DC to non-occupational and nongenetic risk factors (Alser et al. 2020; van den Berge et al. 2023). Likewise CTS, there is a general consensus that the etiology of DC is multifactorial with varying contributions of occupational and individual risk factors which may be due to differences in study design, diagnostic criteria (mainly for mild conditions), methods of ascertainment and measurement of risk factors (qualitative or quantitative), associated musculoskeletal comorbidities, or characteristics of the surveyed populations (differences in genetic, environment and working conditions).

By summing up, the findings of our pooled epidemiological studies revealed that some personal risk factors can concur with HTV exposure to the occurrence of upper limb disorders in users of vibratory tools: (i) older age was a significant predictor of most of the HAVS disorders investigated in the study populations; (ii) metabolic dysfunctions were associated with vascular and fibroproliferative disorders in the fingers and hands; (iii) traumas and/or surgery of the neck/upper limbs were predictive of sensory nerve disorders and CTS; (iv) excessive alcohol intake was associated with an increased risk for CTS and DC; (v) disorders of the cervical spine was related to peripheral sensorineural disturbances, although this finding was observed only in the pooled cross-sectional study. Clinical and laboratory investigations suggested a protective effect of BMI on vascular disorders, confirming the findings of previous epidemiological studies of Raynaud’s phenomenon in the general population and worker groups. Data modelling did not show significant interactions between HTV exposure and individual characteristics suggesting that occupational and personal risk factors may play independent roles in the occurrence of upper limb disorders. We recognise that the lack of statistical significance for interactions between variables does not exclude possible underlying biological synergy between risk factors which can cooperate to the onset and development of adverse health effects in the fingers and hands of HTV exposed workers. However, the presence of personal, non-occupational, risk factors should not be considered a sufficient justification for rebutting the presumption of work relatedness for the disorders in the upper limbs of HTV exposed workers providing that the symptoms and signs of the disorders arise after the first exposure to HTV.

### Limitations

The limitations of the cross-sectional and cohort studies which were combined in the pooled databases have been discussed in details in other papers which the reader may refer to (Bovenzi 1998, 2008, 2010; Bovenzi et al. 1995, 2004, 2011a, b, 2015). Briefly, it is recognised that several sources of bias can affect cross-sectional studies, among which the healthy worker effect, selection bias and information bias. On planning the studies, efforts were made to investigate all active HTV workers employed in the surveyed factories or companies, although selection bias due to work force turnover, retirement, or migration to other jobs of workers affected with HTV related disorders could not be excluded. In the cohort studies, about 86% of the HTV workers participated in at least one follow-up investigation. The main reasons for dropping out of the cohort studies were retirement and change of residence. Only a minority of the HTV workers (about 7%) refused to participate in the follow-up or could not be identified after the cross-sectional survey. To our knowledge, the two pooled studies are among the larger epidemiological investigations with individualised personal, medical and exposure assessments in which the prevalence and incidence of the components of the HAVS and their relations to HTV exposure and individual risk factors were investigated by using validated diagnostic tools for case definitions and recognised quantitative methods to evaluate occupational exposure to vibration. With regard to this latter issue, it is to recognise that some potential sources of uncertainty could affect the estimation of daily vibration exposure. Vibration was measured on the hand-held tools used by the vibration exposed workers at the time the original studies were conducted, and this may be a source of bias since only current vibration exposure was evaluated. However, the frequency weighted rms acceleration magnitudes of vibration measured in the tools were consistent with those reported in recent and past investigations and in vibration guidelines (Griffin et al. 2006). In the original studies, daily duration of exposures to vibration were quantified by means of questionnaire data or direct interview of employees and employers, so that recall bias cannot be excluded. Several investigations have reported that HTV workers tend to overestimate their actual daily duration of exposure to the vibration from tools (Palmer et al. 2000; Gerhardsson et al. 2005). To reduce, at least partially, this bias, in the VIBRISKS cohort study a survey was conducted in the field over an entire week period by supervisors who used a stopwatch method recommended by the EU guide on HTV to measure the time the hands of the worker are actually in contact with the operating tool (Griffin et al. 2006). When a worker operated two or more different power tools, partial vibration exposures were determined from the magnitude and duration of representative operations performed with each tool, and then, the overall daily vibration exposure was calculated according to international standard ISO 5349–2 (2001) and the EU guidance (Griffin et al. 2006).

The possible effects of psychosomatic/psychological strain symptoms on upper limb disorders were investigated only in the HTV workers of the VIBRISKS cohort study and, as a result, these symptoms were not included in the pooled data. We recognised this limitation even though the role of personal psychological symptoms and/or adverse occupational psychosocial factors in the occurrence of CTS and musculoskeletal disorders of the neck and upper limbs is still debated (van den Heuvel et al. 2005; Harris-Adamson et al. 2013). The individual risk factors investigated in the pooled studies included some personal characteristics and comorbidities. The inquired conditions represent a non-exhaustive selection of individual, non-work related, risk factors which could potentially contribute to the occurrence of upper limb disorders when concomitant with HTV exposure. The assessment of personal risk factors and comorbidities was based on self-reporting, and, consequently, recall bias or misunderstanding of disease definition or meaning cannot be excluded; nevertheless in several circumstances, comorbidities could be documented since the patients exhibited medical certificates, reports or prescriptions issued by either their GPs or the consultants of the local Units of the NHS. As aforementioned, cross-sectional studies suffer from several limitations compared to prospective cohort studies. Unfortunately, most of the epidemiological studies of HTV related disorders are of cross-sectional nature. The exposure–response relationship for HTV induced vascular disorders (VWF) reported in international standard ISO 5349-1 (2001) is also based on cross-sectional investigations. Since similar associations were observed between health outcomes and HTV exposure and personal risk factors in our pooled cross-sectional and cohort studies, it may be argued that the findings of cross-sectional investigations can anyway provide useful, although limited, epidemiological data to outline the relations between adverse health outcomes and exposures to HTV and individual risk factors.

## Conclusions

The findings of the pooled analysis of epidemiological studies with individual-level data confirmed that occupational exposure to HTV is a primary risk factor for the occurrence of disorders in the upper limbs of professional users of vibratory tools. Ageing and personal risk factors connected to lifestyles and comorbidities were found to concur with HTV exposure to the development of upper limb disorders. Occupational and individual risk factors tended to contribute independently of each other to adverse outcomes in operators of hand-held vibrating machinery. The findings of our epidemiological studies may be helpful for occupational physicians who have the responsibility to manage and update protocols for the health surveillance of vibration exposed workers. Targeted preventative measures at the workplace and programs for health promotion should be implemented to protect vulnerable workers, with particular considerations for the elderly workers and those affected with comorbidities which can make them more susceptible to the adverse effects of HTV.

## Data Availability

The data that support the findings of this study are available within the article. The data are not publicly available due to privacy restrictions.
